# Impact of Transgenic *Arabidopsis thaliana* Plants on Herbicide Isoproturon Phytoremediation through Expressing Human Cytochrome P450-1A2

**DOI:** 10.3390/biology9110362

**Published:** 2020-10-27

**Authors:** Ehab Azab, Ahmad K. Hegazy, Adil A. Gobouri, Amr Elkelish

**Affiliations:** 1 Department of Biotechnology, College of Science, Taif University, P.O. Box 11099, Taif 21944, Saudi Arabia; 2 Botany and Microbiology Department, Faculty of Science, Zagazig University, Zagazig 44519, Sharkia, Egypt; 3 Botany and Microbiology Department, Faculty of Science, Cairo University, Giza 12613, Egypt; hegazy@sci.cu.edu.eg; 4 Department of Chemistry, College of Science, Taif University, P.O. Box 11099, Taif 21944, Saudi Arabia; a.gobouri@tu.edu.sa; 5 Botany Department, Faculty of Science, Suez Canal University, Ismailia 41522, Egypt

**Keywords:** phytoremediation, *CYP1A2*, transgenic *A. thaliana*, isoproturon, plant growth

## Abstract

**Simple Summary:**

Isoproturon is one of the best selective herbicide for weed control. Excessive use of herbicides causes many environmental problems. In the present study, phytoremediation of phenylurea isoproturon herbicide using transgenic *A. thaliana* plants expressing human cytochrome P450-1A2 were investigated. Toxic effect of isoproturon on the plant phenotypic characteristics was explored. The results revealed that no harmful effects appeared on *CYP1A2* transgenic plants with high tolerance to isoproturon herbicide applications whereas deleterious effects were observed on the morphological characteristics of the wild type grown in soil under different treatments with isoproturon. The transgenic *A. thaliana* plants expressing P450-1A2 were able to metabolize the phenylurea herbicide isoproturon. Therefore, this method can be determined as a potential bioremediation agent.

**Abstract:**

The excessive use of herbicides is a major cause of many environmental problems. The use of isoproturon herbicide as a weed controller has been a common practice globally. Phytoremediation technology can help in cleaning up polluted areas. In this paper the ability of *CYP1A2* transgenic *A. thaliana* plants in the phytoremediation of isoproturon herbicides has been investigated. We tested the capability of P450-1A2 overexpression on the detoxification and degradation of isoproturon. We explored the toxic effect of isoproturon on the plant phenotypic characteristics, including the primary root length, rosette diameter, and fresh, dry weight for transgenic and wild type *A. thaliana*. The results revealed that no morphological changes appeared on *CYP1A2* transgenic plants with a high tolerance to isoproturon herbicide applications either via foliar spraying or supplementation of the growth medium. Deleterious effects were observed on the morphological characteristics of plants of the wild type grown in soil under different treatments with isoproturon. The transgenic *A. thaliana* plants exhibited a vigorous growth even at high doses of isoproturon treatments. In contrast, the growth of the wild type was significantly impaired with doses above 50 µM isoproturon. The transgenic *A. thaliana* plants expressing P450-1A2 were able to metabolize the phenylurea herbicide isoproturon. Therefore, this method can be determined as a potential bioremediation agent.

## 1. Introduction

Ecosystems are increasingly affected by contamination from herbicides, which are frequently used as weed controllers in various agricultural activities. Herbicides can enhance food production by increasing crop yields to satisfy the current needs of the growing world population. However, apart from the benefits, the rapid build-up and the intensive use of toxic herbicides can be detected outside the application areas, causing major environmental hazards [1].

Different phenylurea herbicides—such as linuron, chlorotoluron, and isoproturon—are heavily utilized to kill or inhibit the growth of weeds [2]. The use of the non-ionic herbicide isoproturon C_12_H_18_N_2_O (IPU) has been a common practice globally [3].

The isoproturon (N-(4-isopropylphenyl)-N’, N’’-dimethylurea) is one of the best selective herbicide for the grass and broadleaf pre and post emergence weed control and normally wide spread with many crops, for instance wheat, cereal, sugarcane, and citrus [2,4]. It is widely used in many countries. Isoproturon is registered for use in countries such as India and European Union members [2]. Lebailly et al. [5] reported that, in France, isoproturon is the most heavily used herbicide on wheat and barley. Contamination by isoproturon impacts human health and allows for certain environmental pollutions [6]. It affects the environment adversely, in particular aquatic invertebrates, algae, and microbes [4,7]. Around 3.0 million hectares of agricultural land in the UK (UK) were deposited on approximately 3300 tons of isoproturon in 1997. Isoproturone was found to be contaminating in rivers, streams, coastal waters, and groundwaters as a result of its systematic use [8]. Owing to its effects on the environment, this compound should be extracted from wastewater. Isoproturon is reported as a genotoxic chemical compound and raises concerns regarding its endocrine-disrupting properties. It also poses risks to aquatic life [9]. Behera and Bhunya [10] stated that the administration of isoproturon in adult mice provoked several forms of chromosome aberrations. They emphasized that the highest dosage of isoproturon led to the formation of micronuclei in cells of the bone marrow. These results were agreed with by Srivastava and Raizada [11] where high concentrations of isoproturon in pregnant rats also displayed chromatid breaks in bone marrow cells. Concerning human health, Isoproturon is graded as a Class 2 carcinogenic. The results of reproductive toxicity studies showed that xenobiotic isoproturon may be a disruptive compound in mammals [12].

For environmental protection, extensive treatments are necessary to reduce or dispose of different toxic herbicides. Disposing of contaminants by using the traditional physical and chemical treatments is an improper solution because it is expensive [13]. Although a variety of isoproturon degrading soil micro-flora has been isolated from contaminated sites, for instance *Sphingomonas* sp., *Methylopila* sp., and *Pseudomonas aeruginosa* strain JS-11 were found promising for complete mineralization. Supplementation of carbon to polluted sites will stimulate microbial growth and improve the rate of degradation of complex organic pollutants [14]. However, absence of electron donors, scarcity of nutrients, or slow stimulation of the catabolic pathways acts as limiting factors for pesticide degradation [15]. At the same time, phytoremediation is a highly recommended treatment used to dispose of environmental pollutants [16]. Phytoremediation is considered an effective green technology and low-cost solution for water and soil contamination. There are numerous kinds of phytoremediation methods, such as rhizodegradation, phytodegradation, phytostabilization, rhizofiltration, phytoextraction, and phytovolatilization.

Each method has a pertinent use; phytoextraction eliminates heavy metals from soils by gathering them in plant biomass [17,18]. While in phytodegradation, plants accumulate and degrade contaminants, and phytostabilization decreases the bioavailability of pollutants through binding or immobilizing them into the soil matrix. In the phytovolatilization, the contaminants are transformed and released into the atmosphere. Finally, in rhizodegradation, the microbial activity of the rhizosphere degrade the pollutants in plant roots, and rhizofiltration removes contaminants by plant roots from aqueous medium [19]. Although much literature was introduced to support this endeavor, further research is still needed to develop and understand the phytoremediation mechanisms. Phytodegradation is also known as phytotransformation and has gained intense interest in disposing of the ubiquitous environmental contaminations with persistent and potentially toxic organic compounds, through using plant species to uptake and degrade pollutants [20].

Human cytochrome P450s enzymes comprise a superfamily, including heme monooxygenases. The metabolism of numerous endogenous substrates is highly dependent on P450s; for instance, most vitamins and the steroid hormones [21,22,23,24]. Cytochrome P450 enzymes are of considerable interest and have special biological applications in biotechnology, medicine, pharmacology, and phytoremediation [25,26]. In the last decade, the power of CYP biocatalysts for the phytoremediation of the environmental contaminants using transgenic plants was rapidly realized [27,28]. Kawahigashi et al. [29] elucidated that the majority of the chlorinated pesticides—including linuron, chlorotoluron, atrazine, and isoproturon—could be effectively being oxidized by the cytochrome P450s. Additionally, strong attributes of cytochrome P450 enabled its full range of applications in medicine.

Transgenic plants are genetically modified plants; their DNA is artificially modified by inserting a gene or genes via genetic engineering techniques [30]. Their future remains unclear as the applications have several positive and negative issues [31]. James and Strand [32] introduced an enlightening review for the latest advances in genetic modifications of plants to intensify the phytoremediation of organic pollutants.

The complete genomic sequence of the flowering *Arabidopsis thaliana* plant is well defined and analyzed; it is considered as an essential model for identifying genes and determining their related functions [33]. The *A. thaliana* plant has certain potentials in the phytoremediation process; it is significantly useful in uptaking and degrading certain organic contaminants [34]. The ability to uptake and degrade high concentration levels of herbicides was adequately investigated in many research studies. Azab et al. [35] investigated that the expression of the human P450-1A2 can enhance the tolerance and detoxification of linuron in *A. thaliana* plants. Kebeish et al. [36] declared the phytoremediation of the herbicide chlortoluron in the transgenic *A. thaliana* plants expressed by the mammalian cytochrome P450-1A2. Azab et al. [37] discussed the phytoremediation process of herbicide simazine by using P450-1A2 transgenic *A. thaliana* plants.

Many other studies have been performed on the phytoremediation mechanism using genetically modified *A. thaliana* plants expressing different gene types. Jang et al. [38] investigated the impacts of *A. thaliana* plants overexpressing PgCYP76B93 in the phytoremediation of the herbicide chlortoluron. Khanom et al. [39] reported the involvement of ginseng-derived CYP736A12 in chlortoluron and isoproturon tolerance when overexpressed in *A. thaliana*. Höfer et al. [40] illustrated that the CYP76C1 gene conferred tolerance to chlorotoluron and isoproturon, when overexpressed in native *A. thaliana* plants. For other essential transgenic plant species, such as tobacco, research topics can be found in [41,42].

To the best of our knowledge, limited works have been introduced regarding the phytoremediation of isoproturon using different transgenic plants, and no work was introduced regarding phytoremediation using *A. thaliana* plants expressing human cytochrome P450-1A2. The goal of this work is to assess the phytoremediation of the isoproturon herbicide by using the transgenic *A. thaliana* plants expressing human cytochrome P450-1A2. We investigated the effect of the overexpression of the P450-1A2 gene on the detoxification, degradation, transformation, and metabolism of isoproturon. The toxic effects of isoproturon on plant phenotypic characteristics, including the fresh weight, dry weight, primary root length, and rosette diameter for both wild type and transgenic *A. thaliana* plants, were analyzed.

## 2. Materials and Methods

### 2.1. Plasmid Constructs, Plant Transformation, and Growth Conditions

The coding sequence of human cytochrome P450-1A2 was first amplified by PCR from a cDNA clone derived from Human using 5’-ATCGCCATGGTATGGCTCTGTTATTAGC-3’and 5’-GTAGTCTAGATCAATTGATGGAGAAGCGC-3’ oligonucleotides. CYP1A2 was cloned into the binary plant expression vector pTRAK, a derivative of pPAM (gi13508478). The scaffold attachment region of the tobacco RB7 gene flanked the expression cassettes (gi3522871), the 3‘UTR of CaMV 35S (pA35S), and 5’ UTR of tobacco leader peptide (TL) as described by [35,36,37]. The nptII cassette of pPCV002 was used for selection of transgenic plants on kanamycin. The enhanced CaMV 35S promoter controls the transcription process. CYP1A2 structure expression cassette and the binary plant expression vector were described by [35,36,37]; and confirmation of the transformation is provided in Supplementary 1a. Seeds of *A. thaliana* were obtained from Leibniz Institute of Plant Genetics and Crop Plant Research (Germany) and TAIR certified. Transformed *A. thaliana* plants were obtained as illustrated by [43] using the method of floral dip. The second generation was grown at 22 °C under short-day growth conditions with a photon flux density of 100 µM m^2^s^−1^ and was used for physiological experiments. 

### 2.2. PCR and Western Blot

According to Chomczynski et al. (1995) [44], the leaves of *A. thaliana* were used for the RNA isolation. Synthesis of the first-strand of cDNA was performed as described by [45]. The details were previously described by [35,36,37] and are also shown in Supplementary 1b,c. Leaf-extracted proteins from the *A. thaliana* plant were used to confirm the production of human *CYP1A2* protein in the selected transgenic plants as previously described by [35,36,37] and also shown in Supplementary 1d.

### 2.3. Evaluation of Isoproturon Phytotoxicity on Plant Growth

Two different methods were applied to evaluate the phytotoxicity of isoproturon on plant growth (Table 1). First, a plate assay was performed to determine both the root and shoot growth of seedlings through horizontal and/or vertical Murashige and Skook agar plates (MS) agar plates [46]. Sterilized seeds of wild type and *CYP1A2* transgenic *A. thaliana* plants were grown on (MS) comprising 0.0, 0.2, 0.5, 1.0, 1.5, 2.0, and 2.5 µM of isoproturon (Sigma Aldrich, Germany). After one month, under short-day growth conditions (8 h illumination and 16 h darkness). The primary root lengths and rosette diameters of the grown seeds were measured (Ex1). The selection of transgenic plants was based on the supplementation of 50 μg/mL kanamycin antibiotic within the MS-agar plates.

Secondly, the seedling plate was transferred to a separated pot containing sterilized peat moss soil, for determination of the effect of isoproturon on the phenotype of the wild and *CYP1A2* transgenic *A. thaliana* plants (Table 1). Foliar application of isoproturon (0, 15, 50, 150, and 250 µM) was performed three times at 4-day intervals (Ex2). For the plant-growth-rate foliar-spray-assay of 10 µM isoproturon after 4 weeks of growth in the soil at short day growth conditions, the plant rosette diameter was recorded at 2-day intervals (Ex3). For the fresh and dry weight of wild type (WT) and *CYP1A2* transgenic *A. thaliana* plants, 6-week-grown plants at short day growth conditions were sprayed with 10 µM isoproturon three times at 4-day intervals (Ex4). The plants were then grown for two more weeks before the measurements (Table 1).

### 2.4. Effect of Isoproturon Treatment on Photosynthetic Pigments

The total photosynthetic pigments for the wild type and the three lines of transgenic *CYP1A2 A. thaliana* (Ex4) were evaluated. The chlorophyll and carotenoid concentrations were measured as described by [36].

### 2.5. Statistical Analysis

All data were expressed as the mean ± standard deviation (SD). Each experiment was conducted in four replicates. The experimental data were analyzed using SPSS-22 statistical software. The data were subjected to ANOVA [47] to test the significance (*p* < 0.05*)* differences between the wild and transgenic lines.

## 3. Results

### 3.1. Transgenic A. thaliana Plants Transformation and Selection

Wild type *A. thaliana* was transformed using an *Agrobacterium tumefaciens* strain GV3101 carrying the expression plasmid pTRAK-*CYP1A2* (Supplementary 1a). Transgenic lines were used for the biochemical analyses and regular RT-PCR testing for *CYP1A2* gene was performed before proceeding to isoproturon application.

### 3.2. Effect of Isoproturon Treatments on A. thaliana Plant Primary Root Lengths and Rosette Diameter

**Figure 1:**
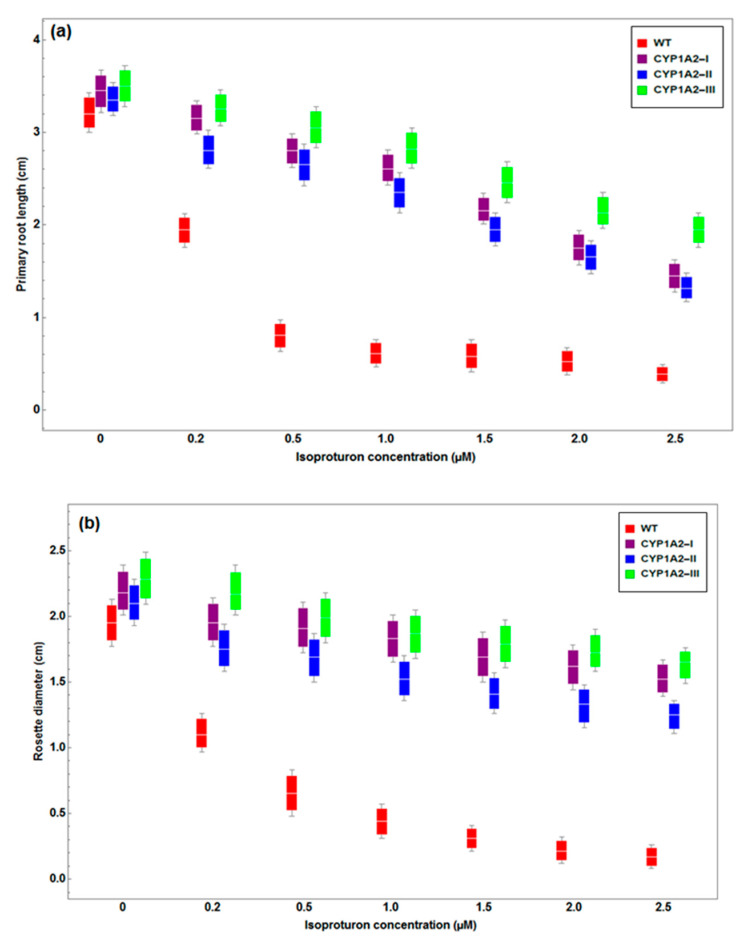
Effect of isoproturon on the primary root length (**a**) and rosette diameter (**b**) of wild type and *CYP1A2* transgenic *A. thaliana* plants grown on Murachige and Skoog agar plates (MS) medium (*p* < 0.05).

The root length and rosette diameter of *A. thaliana* plant exposed to isoproturon treatments in the Ex1 condition are shown in Figure 1. In the wild type, different isoproturon treatments on the MS growth medium showed a harmful effect on the primary root length and rosette diameter. With 2 μM isoproturon concentration, approximately a 60.9% reduction in root length and 56.4% reduction in rosette diameter were observed. There was no significant effect of increasing the isoproturon concentration in transgenic *A. thaliana* lines regarding the root length and rosette diameter (Figure 1). Only gradual retardation on both the root length and rosette diameter was observed for these transgenic plants at higher concentrations of isoproturon above 10 μM. Whereas isoproturon concentrations above 0.2 µM caused significant decrease in the wild type root length and rosette diameter. Figure 1 illustrates that the root length of transgenic plants showed normal growth. In the transgenic plants, regular root extension enables the nutrient uptake, thus, increasing the biomass of the rosette diameter. Our data showed that introducing the mammalian *CYP1A2* gene in plants appeared to enhance its ability to mitigate isoproturon toxicity.

### 3.3. Effect of Isoproturon Treatments on the Total Plant Fresh and Dry Weight

**Figure 2:**
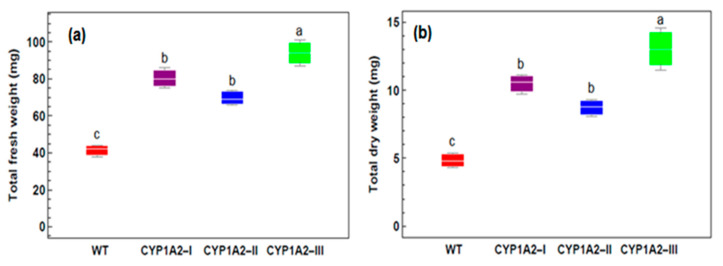
Effects of isoproturon applied by foliar spray on the total fresh weight (**a**), and total dry weight (**b**) of wild type (WT) and *CYP1A2* transgenic *A. thaliana* plants lines. Different lowercase letters indicate significant differences between the wild type and different lines of transgenic *A. thaliana* plants (*p* < 0.05).

The effect of isoproturon on the growth and development of transgenic plants was estimated in the Ex4 condition using the total fresh and dry weight. Figure 2 shows the results of this growth assay. Transgenic *CYP1A2 A. thaliana* plants clarified a definite increase in their fresh and dry weights comparing to the wild type plants after isoproturon application. Transgenic *CYP1A2*-I, *CYP1A2*-II, and *CYP1A2*-III *A. thaliana* plants showed up to 1.04-, 0.75-, and 1.4-fold increases; respectively in the observed fresh weight compared to the fresh weight of the control plants. The same pattern was recorded for the dry weight, which showed up to 1.2-, 0.9-, and 1.73-fold increases, respectively. These findings demonstrate the performance of *CYP1A2* transgenic plants to mitigate the harmful effects of isoproturon on plant growth.

### 3.4. Effect of Isoproturon Treatments on Rosette Diameter of Wild Type and CYP1A2 Transgenic Plants

**Figure 3:**
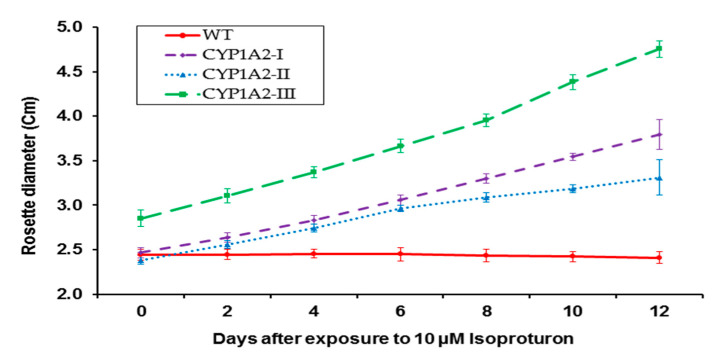
The effects of isoproturon applied by foliar spray on rosettes. WT: wild; *CYP1A2*-I, *CYP1A2*-II, and *CYP1A2*-III: transgenic *A. thaliana* plants *CYP1A2*. (*p* < 0.05).

Wild and transgenic *A. thaliana* plants were allowed to grow in soil until 6–8 rosette leaves appeared. The exogenous application of isoproturon (Ex3) for 12 days was applied, and the rosette diameter was measured before and after isoproturon application. The results in Table 2 show that the wild as well as the transgenic lines had the same growth rate in the untreated plants with isoproturon herbicide, as the growth rate was approximately 1–1.2-fold. Whereas, in the presence of isoproturon, the wild type plants showed complete inhibition of the rosette diameter growth as shown in Figure 3.

In the case of *CYP1A2* transgenic *A. thaliana* plants, an enhancement of the rosette diameter growth was clearly observed after isoproturon foliar application. According to the results in Table 2, the growth pattern of the wild type was inhibited upon isoproturon application. While *CYP1A2*-I, *CYP1A2*-II, and *CYP1A2*-III plants significantly exhibited an increase in their rosette diameter when compared to the wild type. The obtained results clarified that *A. thaliana* plants expressing P450 1A2 were able to metabolize the herbicide isoproturon, which boosted the growth and enhanced their tolerance to isoproturon.

### 3.5. Phenotype of Wild Type (WT) and CYP1A2 Transgenic A. thaliana Plants under Different Concentrations of Isoproturon

**Figure 4:**
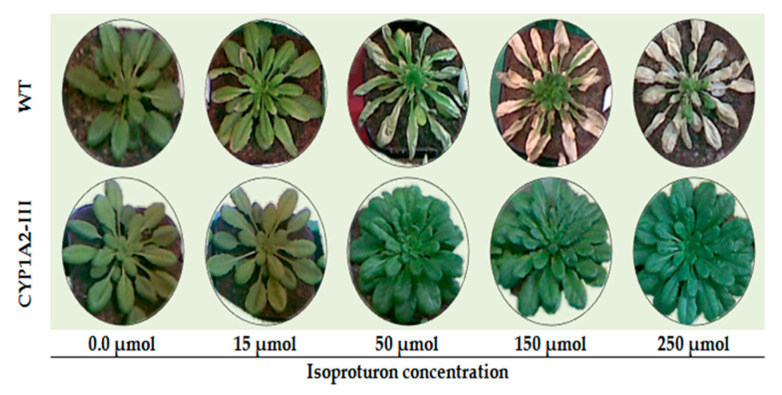
Effects of isoproturon application on the phenotypes of wild type (WT) and *CYP1A2* transgenic *A. thaliana* plants. Wild type (WT) and *CYP1A2*-III transgenic *A. thaliana* plants.

The detoxifying efficiency of isoproturon in wild and transgenic *A. thaliana* (Ex2) was studied and is shown in Figure 4. During the germination test, the harmful effects of isoproturon appeared significantly in the wild type grown in soil with different concentrations. Whereas isoproturon exhibited no harmful effects for the transgenic *A. thaliana* plants as shown in Figure 4.

For the wild type plants, 15 μM isoproturon significantly decreased the plant growth, and increasing the isoproturon above 50 μM exhibited severe damage followed by a final death at 250 μM as shown in Figure 4. In contrast, transgenic *A. thaliana* plants were not affected with even the high concentration of isoproturon. The transgenic plants continued growing, and higher biomass under isoproturon stress was found compared to WT plants.

The phytotoxicity of isoproturon was assessed as LD_50_ for the wild and transgenic *A. thaliana* either in MS medium (Ex1) or in the exogenous applied treatments (Ex2) with variable concentrations of isoproturon (Table 3). In the foliar application treatments (Ex2), the isoproturon concentration at 50% death of *A. thaliana* plants (LD_50_) for the wild type was 28 μM, and, for the transgenic plants *CYP1A2*-I, *CYP1A2*-II, and *CYP1A2*-III, the LD_50_ values were 200, 180, and 250 μM, respectively. While in the growth in MS medium (Ex1), the LD_50_ was 0.35 μM in the WT plants. Whereas the LD_50_ values in the *CYP1A2* transgenic lines increased from 4- to 5.9-fold when compared to the WT plants (Table 3).

### 3.6. Chlorophyll Content of Wild and Transgenic Plants under Isoproturon Treatments

**Figure 5:**
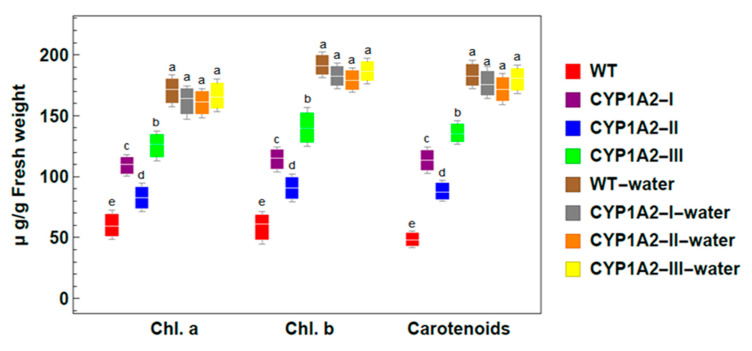
Photosynthetic pigment contents of wild type (WT) and *CYP1A2* transgenic *A. thaliana* plants with and without isoproturon application. Photosynthetic pigments; Chlorophyll a (Chl. a), chlorophyll b (Chl. b), and carotenoid contents. WT: wild type; *CYP1A2*-I, *CYP1A2*-II, and *CYP1A2*-III: *A. thaliana* plants transgenic for the *CYP1A2* gene. Different lowercase letters indicate significant differences between the photosynthetic pigment contents for wild type and different lines of transgenic *A. thaliana* plants (*p* < 0.05).

The effect of isoproturon on the chlorophyll content of wild and transgenic plants (Ex4) is presented in Figure 5. We found a significant reduction in the photosynthetic pigments (chlorophyll a and b and carotenoids) in the wild type after the exogenous application of isoproturon on plants compared to control (wild-water). In contrast, we found a significant induction in the photosynthetic pigments (chlorophyll a and b and carotenoids) after foliar spraying in the transgenic lines (*CYP1A2*) compared to wild type with isoproturon. Subsequently, the transgenic lines exhibited an increase in photosynthetic pigments up to 1.8-fold. Hence, the loss of pigmentation due to isoproturon treatments was clearly decreased by the overexpression of *CYP1A2* in transgenic lines.

The photosynthetic pigments in the transgenic lines were able to withstand and detoxify isoproturon due to the effectiveness of the *CYP1A2* gene in augmenting the effect of the main enzymes associated with the metabolism. This finding demonstrates the ability of transgenic P450 lines to detoxify isoproturon.

## 4. Discussion

Cytochrome P450-dependent isoenzymes are essential metabolic systems in the human body. They have a significant role in metabolizing many drugs and carcinogens [48]. *CYP1A2* is one of the individual P450 species that exhibits an essential role in the biodegradation of several xenobiotics, such as drugs, pesticides, and herbicides [49]. Introducing the *CYP1A2* gene in *A. thaliana* plants provided a new suitable plant species that can biodegrade several kinds of herbicides. In this study, the phytoremediation efficiency of *CYP1A2* transgenic *A. thaliana* plants in degrading the isoproturon herbicide was tested. Several reports investigated the ability of transgenic *A. thaliana* plants to take up and metabolize different environmental organic pollutants [35,37].

Recently, several scientists explored the impact of the mammalian P450 isoenzymes CYP1–CYP3 in higher plants on herbicide and pesticide phytoremediation [29,50,51,52,53]. Our data showed that transgenic *A. thaliana* successfully broke down soil contaminants and depolluted the injurious effects of pesticides and herbicides. This is attributable to cytochrome P450’s crucial role in depredating and metabolizing different organic contaminants [39,54,55].

In our study, the transgenic *CYP1A2* plants displayed no noticeable phenotypic effects on the growth and development of the plant in comparison to the wild type Figure 5. This indicates that the expression of the *CYP1A2* gene did not interfere in any essential pathways or produce undesirable toxic compounds affecting the plant development. After the isoproturon application, *CYP1A2* transgenic plants exhibited a high tolerance to isoproturon in comparison to wild type plants. This refers to introducing the *CYP1A2* gene, which enhances the ability of *A. thaliana* in the metabolization and removal of phenylurea herbicide. Through the expression of mammalian *CYP1A2* genes in transgenic plants, the phytotoxic effects of isoproturon pollutants were overcome, resulting in an increase in the metabolism and removal of different kinds of organic contaminants and herbicides.

Previous studies demonstrated similar results on the biodegradation of other phenylurea herbicides—such as linuron [35], chlortoluron [36], and simazine [37]—using *CYP1A2* transgenic *A. thaliana* plants. The same trend was described by Höfer et al. [40], who illustrated that the CYP76C1 gene conferred tolerance to chlorotoluron and isoproturon when overexpressed in native *A. thaliana* plants. For other transgenic plants, such as tobacco and rice, Bode et al. [56] proved that transgenic tobacco cell cultures expressing human P450 species 1A1 or 1A2 enhanced the bioremediation capability of atrazine herbicide. Kawahigashi et al. [29] illustrated that transgenic CYP1A1 rice plants enhanced the herbicide tolerance toward different phenylurea herbicides, including chlorotoluron, mefenacet, and norflurazon. Didierjean et al. [57] indicated that the CYP76B1 gene, introduced within tobacco and *A. thaliana* plants, improved their ability to metabolize phenylurea isoproturon, chlortoluron, and linuron herbicides.

In this study, after the foliar application of isoproturon herbicide, the *CYP1A2* transgenic *A. thaliana* plants displayed a normal vigor growth, while the non-transgenic plants were extensively injured with a concentration above 50 µmol. This could be due to the damaging effect of the herbicide on the photosynthetic electron transport leading to decreased photosynthetic activity and fresh and dry biomass of the wild type [58]. Javaid et al. [59] determined that, for all isoproturon herbicidal applications, the lowest photosynthetic rate of wheat was recorded with isoproturon at 175 g. In the present work, the recorded LD*_50_* indicated that the transgenic *CYP1A2* plants could metabolize most of the riveted isoproturon with a vigorous morphological appearance. In former reports, the LD*_50_* values recorded for *CYP1A2* transgenic plants were 200 µM with linuron [35], 250 µM with simazine [37], and 210 µM with chlortoluron [36]. Additionally, Didierjean et al. [57] recorded that LD*_50_* values for CYP76B1 transgenic tobacco were 90 µM with isoproturon, 180 µM with chlortoluron, and 220 µM with linuron.

According to the obtained results, transgenic *CYP1A2 A. thaliana* plants showed high efficiency toward isoproturon herbicide degradation and can be recommended for the phytoremediation of correspondingly contaminated sites. Similar effects were detected with the transgenic *CYP1A2* line that displayed outstanding cross-resistance to the photosynthesis-inhibiting herbicide linuron [35], and chlortoluron [36]. Transgenic rice plants expressing the CYP2C19 gene also represented high efficiency toward the mefenacet, metolachlor, and norflurazon herbicides [60]. CYP1A1 transgenic rice revealed excessive effectiveness against the chlorotoluron herbicide and metabolized it more easily compared to non-transgenic plants after its exogenous application [29].

In tolerant soybean plants, isoproturon (IPU) was detoxified to monodesmethyl-IPU, 2-hydroxy-IPU, and 2-hydroxy-monodesmethyl-IPU. While in the case of wheat, the major metabolic pathway degraded the isoproturon (IPU) to 2-hydroxy-IPU as a primary metabolite, then to 2-hydroxy-monodesmethyl-IPU, 2-Hydroxy-IPU, and an olefinic metabolite (isopropenyl-IPU) [61]. Data on the phytotoxic features of the unidentified metabolite found in this study are not clear; however, the resulting metabolic compounds may be less phytotoxic than isoproturon. The non-extractable produced residues may be observed as detoxification products. Therefore, transgenic *CYP1A2 A. thaliana* plants are highly recommended in the detoxification of isoproturon herbicides.

## 5. Conclusions

Herbicides have biological and economic benefits; however, they can accumulate in the biosphere and cause long-term adverse effects on the surrounding environment. Phytoremediation appears to be one of the best available technologies for cleaning up polluted sites. Phytoremediation can either completely remove or at least successfully lower the level of pollutant concentrations. Transgenic plants can help in metabolizing the toxic organic chemicals in polluted areas. Transgenic *A. Thaliana* plants expressing human cytochrome P450s previously showed tolerance activity toward many organic herbicides. In the present study, we assessed the phytoremediation of phenylurea isoproturon herbicide using transgenic *A. thaliana* plants expressing human cytochrome P450-1A2. The transgenic *CYP1A2 A. thaliana* plants were able to metabolize the phenylurea herbicide isoproturon through the enhancement of the absorption and detoxification of isoproturon, thereby aiding in the phytoremediation of the polluted environment.

## Figures and Tables

**Table 1:** Experimental design and treatment condition for the evaluation of isoproturon phytotoxicity on plant growth for wild and *CYP1A2* transgenic *Arabidopsis thaliana* plants.

Phytotoxicity of Isoproturon				
	Plate Experiment	Pot Experiment		
Parameters	Root Length and Rosette Diameter	Phenotype	Growth Rate	Fresh and Dry Weight
Tested stage	Seeds on MS medium	7–8 weeks old	4 weeks old	6 weeks old
Exp. code	Ex1	Ex2	Ex3	Ex4
Isoproturon (µM)	0, 0.2, 0.5, 1.0, 1.5, 2.0, and 2.5	0, 15, 50, 150, and 250	10	10
Frequently	One time	Three times	One time	Three times
Days intervals	Once	4 days	Once	4 days
Duration	28 days	12 days	12 days	12 days
Obtained results	End of the experiment	Photo taken 2 days after last dose	Rosette diameter was recorded at 2 days intervals	Weight taken 14 days after last dose

**Table 2:** Isoproturon effect on the growth rate of *A. thaliana* wild type compared to different transgenic lines (*n =* 4).

*A. thaliana* Plants	WT	*CYP1A2*-I	*CYP1A2*-II	*CYP1A2*-III
Growth Rate (cm/day)	0.00	0.08	0.16	0.14
Without isoproturon fold increase in (RD)	1.1	1.1	1.2	1.0
With 10 μM isoproturon fold increase in (RD)	0.00	0.54	0.39	0.67

Growth rate of wild type compared to transgenic *A. thaliana* plants after isoproturon applications as Ex3. RD: Rosette diameter.

**Table 3:** LD_50_ values of isoproturon in plants.

*A. thaliana* Plants	WT	*CYP1A2*-I	*CYP1A2*-II	*CYP1A2*-III
LD_50_ (growth medium)	0.35 μM ± 0.05	1.90 μM ± 0.21	1.75 μM ± 0.33	2.4 μM ± 0.42
LD_50_ (foliar application)	28 μM ± 3	200 μMol ± 13	180 μMol ± 16	250 μMol ± 18

LD_50_ denotes isoproturon concentrations at 50% death of *A. thaliana* plants depending on different isoproturon treatments. Values are the mean ± SE at (*n = 5*) for each genotype.

## Supplementary Materials

The following are available online at https://www.mdpi.com/2079-7737/9/11/362/s1, Supplementary 1. *CYP1A2* gene expression cassette (A), PCR (B), Real Time RT-PCR (C), and Western blot analyses of transgenic *A. thaliana plants* (D).
