# Supplementary material for: Impact of Transgenic Arabidopsis thaliana Plants on Herbicide Isoproturon Phytoremediation through Expressing Human Cytochrome P450-1A2
Source: Biology (Basel). 2020 Oct 27;9(11):362. doi: 10.3390/biology9110362 (PMC7692224; doi:10.3390/biology9110362)
Supplement: Supplementary file 1 [file biology-09-00362-s001.pdf]

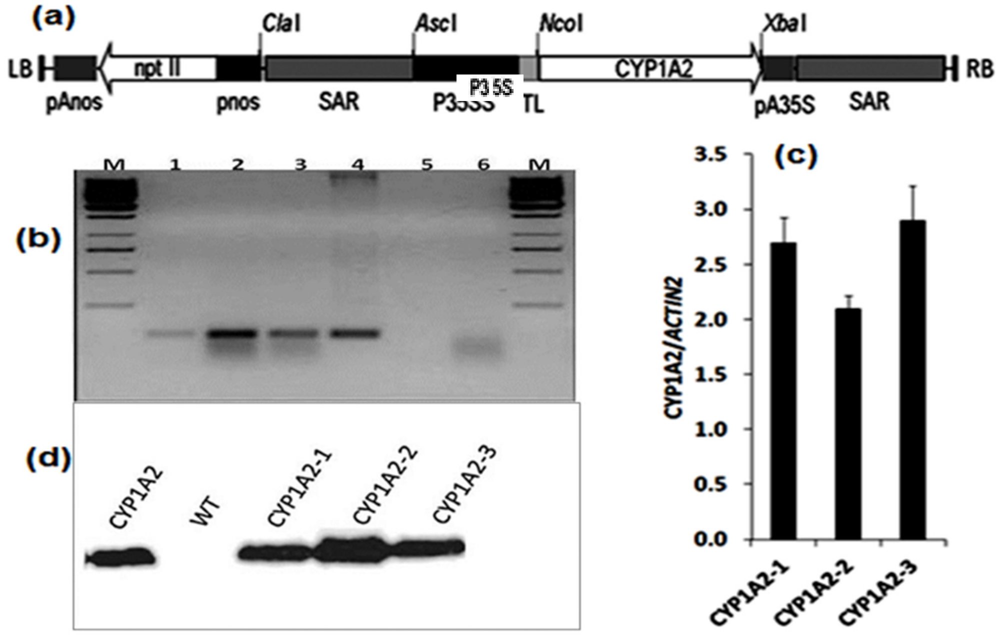

Supplementary 1. *CYP1A2* gene expression cassette (A), PCR (B), Real Time RT-PCR (C), and Western blot analyses of transgenic *A. thaliana plants* (D). (A) The binary plant expression vector pTRAK, a derivative of pPAM (gi13508478) containing a constitutive CaM promoter (p3 5S) and the 50 UTR of the Tobacco Leader peptide (TL) was used for the expression of *CYP1A2* gene in A. thaliana plants. (B) PCR screening results of selected *CYP1A2* transgenic *A. thaliana* plants after 1% DNA agarose gel electrophoresis. Lane 1: positive control using pTRA-K-TL-CYP1A2 as template, Lane 2, 3, and 4: CYP1A2 PCR products using transgenic A. thalina plants DNA, Lane 5: WT A. thaliana plants DNA, Lane 6: negative control. (C) Reverse transcriptase RT-PCR analysis of *CYP1A2* gene expression in transgenic A. thaliana plants. (D) Western blot analysis showing the presence of human *CYP1A2* protein in transgenic lines. PTRAKCYP1A2: the binary plant expression plasmid carrying *CYP1A2* gene sequence. WT: wild type, *CYP1A2-1, 2*, and 3: three independent *A. thaliana* plants transgenic for the human *CYP1A2* gene. Azab et al [35, 37].
